# Public health response to two imported, epidemiologically related cases of Lassa fever in the Netherlands (ex Sierra Leone), November 2019

**DOI:** 10.2807/1560-7917.ES.2020.25.15.2000265

**Published:** 2020-04-16

**Authors:** Femke Overbosch, Mark de Boer, Karin Ellen Veldkamp, Pauline Ellerbroek, Chantal P Bleeker-Rovers, Bram Goorhuis, Michele van Vugt, Annemiek van der Eijk, Tjalling Leenstra, Martin Khargi, Jeanette Ros, Diederik Brandwagt, Manon Haverkate, Corien Swaan, Chantal Reusken, Aura Timen, Marion Koopmans, Jaap van Dissel, Lassa fever response team of The Netherlands

**Affiliations:** 1 Stichting BeroepsOpleiding Huisartsen (SBOH), Utrecht, the Netherlands; 2 Department of Infectious Diseases, Leiden University Medical Center, Leiden, the Netherlands; 3 Department of Medical Microbiology, Leiden University Medical Center, Leiden, the Netherlands; 4 Department of Infectious Diseases, University Medical Center Utrecht, Utrecht, the Netherlands; 5 Department of Internal Medicine, Division of Infectious Diseases, Radboud university medical center, Nijmegen, the Netherlands; 6 Centre of Tropical Medicine and Travel Medicine, Amsterdam University Medical Center, location AMC, Amsterdam, the Netherlands; 7 Department of Viroscience, Erasmus University Medical Center, Rotterdam, the Netherlands; 8 Department of Infectious Diseases, Public Health Service Amsterdam, Amsterdam, the Netherlands; 9 Department of Infectious Diseases, Public Health Service Hollands Midden, Leiden, the Netherlands; 10 Department of Infectious Diseases, Public Health Service Kennemerland, Haarlem, the Netherlands; 11 Department of Infectious Diseases, Public Health Service region Utrecht, Zeist, the Netherlands; 12 National Institute for Public Health and the Environment (RIVM), Centre for Communicable Disease Control (CIb), Bilthoven, the Netherlands; 13 Athena Institute, VU University Amsterdam, the Netherlands; 14 The members of the Lassa fever response team of the Netherlands have been listed at the end of this article

**Keywords:** Lassa fever, health care workers, the Netherlands, public health measures

## Abstract

On 20 November 2019, Lassa fever was diagnosed in a physician repatriated from Sierra Leone to the Netherlands. A second physician with suspected Lassa fever, repatriated a few days later from the same healthcare facility, was confirmed infected with Lassa virus on 21 November. Comprehensive contact monitoring involving high- and low-risk contacts proved to be feasible and follow-up of the contacts did not reveal any case of secondary transmission in the Netherlands.

Two patients with Lassa haemorrhagic fever were diagnosed following nosocomial exposure in a hospital in the district Tonkolili in Sierra Leone. We aimed to reconstruct the measures undertaken regarding the patients and their contacts, identify the lessons learned and formulate recommendations for future cases of importation of patients with haemorrhagic fever. 

## Epidemiological description of the case patients

Both cases – Case 1 (C1) and Case 2 (C2) - had been working in a rural hospital in the Tonkolili district in Sierra Leone. On 4 November 2019, two Dutch healthcare workers (C1 and C2) and one local (C3) participated in obstetric surgical procedures in two local patients who were later presumed to be the source of Lassa virus (LASV) infection. One patient died on the day of surgery after resuscitation during which C1 was not wearing optimal personal protective equipment (PPE). The other patient died 2 weeks later. Haemorrhage was reported in both patients.

On 11 November, while attending an international course in Freetown, C1 started to develop non-specific symptoms (headache, muscle ache, arthralgia, fever, diarrhoea, vomiting and cough). C1 was treated locally for the most probable tropical diseases (such as malaria and typhoid fever).

After 8 days of persisting symptoms, C1 was medically evacuated to the Netherlands on 19 November on a commercially run private plane with a German flight crew, with a transit in Morocco. No specific infection precautions were taken on the flight. C1 was initially transferred by ambulance (Ambulance 1) to the Amsterdam University Medical Center (location AMC, Hospital 1), also without specific infection precautions. Staff in the hospital used MRSA (meticillin-resistant *Staphylococcus aureus*) airborne strict isolation measures and PPE (gloves, FFP2 masks and gowns). Upon suspicion of Lassa fever, C1 was relocated in a dedicated ambulance (Ambulance 2) to the Leiden University Medical Center (LUMC, Hospital 2) dedicated facility for treatment of highly contagious infections. The diagnosis of Lassa fever was confirmed on 20 November by RT-PCR and genome sequencing performed at Erasmus Medical Center (EMC) in Rotterdam. After rapid clinical deterioration, the patient died on 23 November. Stringent hygienic precautions were taken for management of the corpse.

C2 also started to develop non-specific symptoms (fever, vomiting and anorexia) on 11 November and was unsuccessfully treated in Sierra Leone for the most probable tropical diseases. RT-PCR on plasma samples of C2, sent to the EMC, tested positive for LASV on 21 November at and the decision was made for medical evacuation to the Netherlands. In a clinically stable condition, C2 was airlifted on 23 November under strict isolation measures by a French flight crew of Airlec Medical. C2 was transported in a dedicated ambulance (Ambulance 3) to the Major Incident Hospital at the University Medical Centre Utrecht (UMCU, Hospital 3), and admitted to a facility for highly contagious infections. C2 was discharged on 12 December, after two negative results within an interval of 48 h in serum tests for presence of LASV RNA. The patient was discharged into home isolation; as LASV RNA remained positive in the urine, strict instructions regarding hygiene were enforced until urine tested negative after 12 days. 

C3 was a local healthcare worker who was confirmed with Lassa fever infection by the authorities in Sierra Leone. The case history and contact tracing around this case are not part of this report.

## Contact tracing

Contact tracing was initiated upon confirmation of the diagnosis in C1 as viral haemorrhagic fevers are mandatorily notifiable according to Dutch law [1]. Immediately, a response team convened at the Centre for Communicable Diseases (CIb), consisting of representatives of the hospitals, the reference laboratory (EMC), involved public health services (PHS 1–5), ambulance services and experts from the CIb. The response team provided scientific advice on the risk assessment, risk classification and control measures regarding contacts and coordinated the risk communication [2,3].

The contacts of C1 and C2 (including all transportation and hospital staff) were interviewed to assess the intensity of exposure to the cases. All Dutch healthcare workers repatriated from Sierra Leone and the ones who were contacts of the presumed source patients were listed. Contacts were classified into three risk groups according to the nature of their exposure (Table). The control measures were targeted to each risk level, a procedure validated in a previous case [4,5].

**Table:** Dutch risk classification of contacts exposed to healthcare workers with RT-PCR-confirmed Lassa fever contracted in Sierra Leone, including numbers of contacts inventoried in the Netherlands, December 2019

Type of contact	Risk	Mandatory measures until 21 days post exposure	Number of contacts in the Netherlands
High-risk contacts^a^	Contact with patient or body fluids without appropriate PPE	- Temperature check 2×/day - Daily contact with public health service or hospital staff - Prohibition to travel abroad - Work restrictions - Safe sex (condom use)	19 (Hospital 1, Ambulance 1, friends SL, colleagues SL, family)
Low-risk contacts^a,b^	Contact with patient or body fluids with use of appropriate PPE	Temperature check 2×/day	131 (Hospital 1–4, ambulance 2–3, family)
Sporadic contacts	Presence in same room without direct contact	No risk, no measures	14 (Hospital 1)

PPE: personal protective equipment, SL: Sierra Leone.

^a^ In case of fever ≥ 38 °C measured twice,12 h apart, contacts are instructed to consult their assigned healthcare worker (municipal health service or hospital staff) and to avoid new contacts.

^b^ Including contacts without direct contact, but who have been working in and around the hospital in Sierra Leone.

## Contact monitoring

In total, 164 contacts who (temporarily) resided in the Netherlands were identified for follow-up. Nineteen were classified as high-risk contacts [6] (Figure). Monitoring of high- and low-risk contacts, respectively, ended on 15 December and 2 January 2020, 21 days after the last exposure (Table). Post-exposure prophylaxis (PEP) was not prescribed to contacts in the Netherlands. Two contacts (one high- and one low-risk) developed a fever, but an acute LASV infection was excluded in EDTA-plasma with RT-PCR. All high-risk contacts were considered as non-infected as paired serum samples taken at the beginning and at the end of the tracing of high risk contacts revealed no seroconversion for LASV-specific IgM and IgG by both immunofluorescence assay and ELISA (Bernhard Nocht Institute, Hamburg, Germany).

**Figure:**
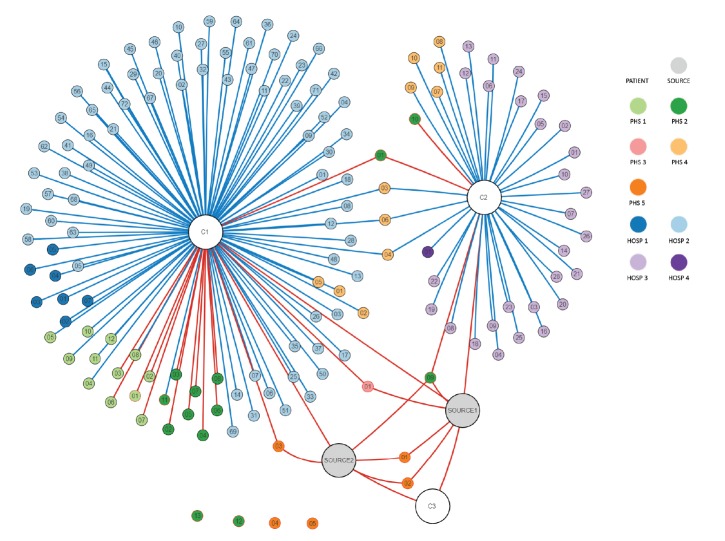
Contact tree representing the high- and low-risk contacts in the Netherlands of the assumed source patients in Sierra Leone and of two Dutch healthcare workers diagnosed with confirmed Lassa fever, the Netherlands, December 2019 (n = 150)

Hosp: hospital; PHS: Public Health Service.

Large grey spheres: assumed source patients with Lassa fever in the local hospital, Sierra Leone. Large white spheres: confirmed secondary cases (C1, C2 and another healthcare worker (C3) involved in the surgical procedures of the assumed source patients). Small spheres: contacts. Red line: high-risk contact, blue line: low-risk contact, no line: contact without direct contact, but followed up as low-risk as they have been working in and around the hospital in Sierra Leone. Red outer line of sphere: contact returned from Sierra Leone, black outer line of sphere: contact in the Netherlands.

Dutch hospitals involved: AUMC location AMC (Hosp 1), LUMC (Hosp 2), UMCU (Hosp 3) and EMC (Hosp 4). Dutch Public Health Services involved: Public Health Service Hollands Midden (PHS 1), Amsterdam (PHS 2), Rotterdam-Rijnmond (PHS 3), Kennemerland (PHS 4), and region Utrecht (PHS 5).

The figure displays the confirmed cases (C1, C2, C3), the presumed source patients and those contacts that were followed up in the Netherlands. The figure does therefore not display: the 14 sporadic cases identified in the Netherlands, the high- and low-risk contacts identified elsewhere in the European Union, the German and French flight crews, the local and Dutch contacts of C1 and C2 who resided in Sierra Leone and the local contacts and secondary cases of the presumed source patients who resided in Sierra Leone.

Dedicated air transportation was arranged for five Dutch high-risk contacts and British contacts who still resided in Sierra Leone. Other contacts in Europe were identified, according to the stepwise backwards contact tracing starting with the air ambulance, in Germany (n = 5), the United Kingdom (UK) (n = 18), Denmark (n = 5) and Norway (n = 2).These contacts have been followed up by the corresponding national authorities, but further spread has not been reported.

Communication with a representative of the hospital in Sierra Leone was established and criteria were exchanged for the identification and monitoring of persons who had potentially been exposed locally. As the contact investigation had revealed contacts from other countries, authorities in Germany, the UK, Denmark and Norway were informed between 20 and 27 November using the Early Warning and Response System (EWRS) of the European Union (EU). On 20 November, an official notification was issued through the EWRS and the World Health Organization Event Information Site. The International Health Regulations National Focal Points of Sierra Leone and Morocco were officially informed by the Dutch authorities.

## Discussion

Lassa virus is a single-stranded RNA virus belonging to the family *Arenaviridae*. It is endemic in several West African countries, in particular Sierra Leone, Liberia, Guinea and Nigeria, although cases had been reported only sporadically in the Tonkolili region [7,8]. Rodents act as a reservoir and shed the virus in urine and droppings. Humans become infected through contact with contaminated rodent excreta, e.g. via objects or inhalation of aerosols. Human-to-human transmission is primarily nosocomial through patients’ body fluids or contaminated fomites when PPE is not in place [5].

Lassa virus causes an estimated 300,000 infections per year worldwide with, in 80% of the cases, no or very mild symptoms and, in 20% of cases, severe disease (haemorrhages and multi-organ failure) [6,9]. The case fatality is 15–20% in hospitalised cases [10]. There is no evidence of human-to-human transmission from asymptomatic carriers, but well-designed studies to address this question are lacking [9,11].

This report shows that LASV can pose an infection risk during routine invasive hospital procedures involving patients in endemic areas, in particular on maternity wards as LASV has a high affinity for placenta and vascular tissues [7,12]. Awareness of the local risks and implementation of standard precautions to reduce the risk of transmission of blood-borne pathogens are essential to prevent nosocomial transmission [8,13]. As LASV infection is initially difficult to diagnose clinically, rapid and accurate differential laboratory diagnostics are crucial to initiate appropriate supporting care and to set up measures to prevent human-to-human transmission [9,10].

PEP with ribavirin was not advised for contacts in the Netherlands because the evidence on effectiveness is inconclusive while potential side effects can be severe [14,15]. Favipiravir and experimental monoclonal antibodies (which have shown encouraging results in animal models) were procured, to be used upon clinical indication [16].

The psychosocial burden of the death of the Dutch healthcare worker and of the measures on the patients, contacts and their families was reported as considerable. Protocols are required that adequately balance the necessary containment measures and the psychosocial burden on patients, contacts and care providers [10].

The response teams in the involved countries in the EU and European Economic Area appeared to use different Lassa fever protocols regarding PEP and testing of asymptomatic contacts (personal communication, UK EWRS team, 23 November 2019). There is a clear need for evidence-based practices implemented in standardised policies across countries.

## References

[1] National Coordination Centre for Communicable Disease Control (LCI). Virale hemorragische koorts – arenavirussen. [Viral haemorrhagic fevers caused by arenaviruses]. Bilthoven: Rijksinstituut voor Volksgezondheid en Milieu; 2019. Dutch. Available from: https://lci.rivm.nl/richtlijnen/virale-hemorragische-koorts-arenavirussen

[2] Kraaij-Dirkzwager M Timen A Dirksen K Gelinck L Leyten E Groeneveld P Middle East respiratory syndrome coronavirus (MERS-CoV) infections in two returning travellers in the Netherlands, May 2014. Euro Surveill. 2014;19(21):20817. 10.2807/1560-7917.ES2014.19.21.20817 24906375

[3] Timen A Koopmans MP Vossen AC van Doornum GJ Günther S van den Berkmortel F Response to imported case of Marburg hemorrhagic fever, the Netherland. Emerg Infect Dis. 2009;15(8):1171-5. 10.3201/eid1508.090015 19751577 PMC2815969

[4] van Steenbergen J Wijnands S Public health management of fatal case of Lassa fever in the Netherlands. Euro Surveill. 2000;4(31):1552.

[5] Veldkamp PJ Schippers EF Een man met fatale Lassa-koorts na een verblijf in Sierra Leone. [A man with fatal Lassa fever following a stay in Sierra Leone.] Ned Tijdschr Geneeskd. 2002;146(46):2201-4. Dutch. 12467165

[6] World Health Organization (WHO). Lassa fever. Fact sheet. Geneva: WHO; 2017. http://origin.who.int/mediacentre/factsheets/fs179/en/

[7] Okogbenin S Okoeguale J Akpede G Colubri A Barnes KG Mehta S Retrospective cohort study of Lassa fever in pregnancy, Southern Nigeria. Emerg Infect Dis. 2019;25(8):1494-500. 10.3201/eid2508.181299 31310586 PMC6649346

[8] World Health Organization (WHO). Lassa fever – the Netherlands (ex –Sierra Leone). Geneva: WHO; 2019. Available from: https://www.who.int/csr/don/27-november-2019-lassa-fever-netherlands_sierra_leone/en/

[9] Garnett LE Strong JE Lassa fever: With 50 years of study, hundreds of thousands of patients and an extremely high disease burden, what have we learned? Curr Opin Virol. 2019;37:123-31. 10.1016/j.coviro.2019.07.009 31479990

[10] Kofman A Choi MJ Rollin PE Lassa fever in travelers from West Africa, 1969-2016. Emerg Infect Dis. 2019;25(2):245-8. 10.3201/eid2502.180836 30666924 PMC6346466

[11] Ogoina D Lassa fever: A clinical and epidemiological review. Niger Delta J Med Med Res. 2013;1(1):1-10.

[12] Agboeze J Nwali MI Nwakpakpa E Ogah OE Onoh R Eze J Lassa fever in pregnancy with a positive maternal and fetal outcome: A case report. Int J Infect Dis. 2019;89:84-6. 10.1016/j.ijid.2019.08.023 31465848

[13] World Health Organization (WHO). Interim infection prevention and control guidance for care of patients with suspected or confirmed filovirus haemorrhagic fever in health-care settings, with focus on Ebola. Geneva: WHO; 2014. Available from: https://apps.who.int/iris/handle/10665/130596

[14] Bausch DG Hadi CM Khan SH Lertora JJ Review of the literature and proposed guidelines for the use of oral ribavirin as postexposure prophylaxis for Lassa fever. Clin Infect Dis. 2010;51(12):1435-41. 10.1086/657315 21058912 PMC7107935

[15] Eberhardt KA Mischlinger J Jordan S Groger M Günther S Ramharter M Ribavirin for the treatment of Lassa fever: A systematic review and meta-analysis. Int J Infect Dis. 2019;87:15-20. 10.1016/j.ijid.2019.07.015 31357056

[16] Cross RW Hastie KM Mire CE Robinson JE Geisbert TW Branco LM Antibody therapy for Lassa fever. Curr Opin Virol. 2019;37:97-104. 10.1016/j.coviro.2019.07.003 31401518

